# Household and Individual Risk Factors for Cholera among Cholera Vaccine Recipients in Rural Haiti

**DOI:** 10.4269/ajtmh.16-0407

**Published:** 2017-05-30

**Authors:** Wilfredo R. Matias, Jessica E. Teng, Isabelle J. Hilaire, Jason B. Harris, Molly F. Franke, Louise C. Ivers

**Affiliations:** 1 Department of Global Health and Social Medicine, Harvard Medical School, Boston, Massachusetts;; 2 Division of Global Health Equity, Brigham and Women’s Hospital, Boston, Massachusetts;; 3 Partners In Health, Boston, Massachusetts;; 4 Zanmi Lasante, Pont-Tambour, Impasse Guillaume No. 6, Saint Marc, Haiti;; 5 Division of Infectious Diseases, Massachusetts General Hospital, Boston, Massachusetts;; 6 Department of Pediatrics, Harvard Medical School, Boston, Massachusetts

## Abstract

Oral cholera vaccination was used as part of cholera control in Haiti, but the vaccine does not provide complete protection. We conducted secondary data analyses of a vaccine effectiveness study in Haiti to evaluate risk factors for cholera among cholera vaccine recipients. Individuals vaccinated against cholera that presented with acute watery diarrhea and had a stool sample positive for *Vibrio cholerae* O1 were included as cases. Up to four vaccinated individuals who did not present for treatment of diarrhea were included as controls for each case, and matched by location of residence, enrollment time, and age. We evaluated sociodemographic characteristics and risk factors for cholera. Univariable and multivariable logistic regression were performed to identify risk factors for cholera among vaccinees. Thirty-three vaccine recipients with culture-confirmed cholera were included as cases. One-hundred-and-seventeen of their matched controls reported receiving vaccine and were included as controls. In a multivariable analysis, self-reporting use of branded household water disinfection products as a means of treating water (adjusted relative risk [aRR] = 44.3, 95% confidence interval [CI] = 4.19–468.05, *P* = 0.002), and reporting having a latrine as the main household toilet (aRR = 4.22, 95% CI = 1.23–14.43, *P* = 0.02), were independent risk factors for cholera. Self-reporting always treating water (aRR = 0.09, 95% CI = 0.01–0.57, *P* = 0.01) was associated with protection against cholera. The field effectiveness of water, sanitation, and hygiene interventions used in combination with cholera vaccination in cholera control should be measured and monitored over time to identify and remediate shortcomings, and ensure successful impact on disease control.

## INTRODUCTION

Cholera remains a significant cause of morbidity and mortality worldwide, mainly affecting regions that do not have the population-level water, sanitation, and hygiene (WASH) infrastructure that eliminated the disease in Europe and North America over a century ago.^1^ Oral cholera vaccines (OCVs) are increasingly being deployed as part of a comprehensive approach to prevent cholera globally, and multiple studies have demonstrated their effectiveness.^2^ However, they do not provide complete protection; vaccinated individuals can still contract the disease.^3–5^ As such, understanding risk factors for cholera among populations that have been vaccinated against cholera is of critical importance to determine how to design comprehensive integrated programs to eliminate transmission of cholera in the near term.

Risk factors for infectious diseases such as cholera vary in space and time, depending on the social, environmental, and biological contexts in which they occur.^6,7^ Cholera outbreaks have been attributed to population-level risk factors including climate conditions,^8^ the presence of specific copepod hosts,^8^ and human migration patterns, among others.^9^ Individual-level risk factors for cholera have also been well-described and include inadequate WASH,^10^ ingestion of contaminated food or beverages,^11^ and host characteristics (i.e., blood group O, retinol deficiency).^12,13^ Previous studies have demonstrated how population-level immunity can modulate other risk factors for cholera, such as the risk associated with weather fluctuations,^14^ and how cholera vaccination campaigns can improve knowledge and practices related to diarrheal disease^15^; these suggest that vaccination may modulate biological, behavioral, and environmental susceptibility to cholera. However, to our knowledge, no study has examined the specific factors that contribute to cholera disease among vaccinated individuals.

Currently, there are three available OCVs prequalified by the World Health Organization (WHO): Dukoral^®^ (SBLVAccin, Stockholm, Sweden), Shanchol^®^ (Shantha Biotechnics, Hyderabad, India) and Euvichol^®^ (Eubiologics, Seoul, South Korea).^2,16^ A stockpile containing millions of doses of OCV for deployment and distribution to cholera epidemic and endemic areas, including Haiti, was created in 2013 by the WHO.^17^ As the use of OCV continues to expand globally, an understanding of modifiable risk factors among vaccine recipients will inform which nonimmunologic interventions, in particular WASH approaches, might best be used to complement OCV vaccination campaigns in the immediate and near term, when epidemics are underway.

In this context, we present the first assessment of risk factors for cholera among OCV recipients, identified through secondary analyses of a field-based case-control study to evaluate OCV effectiveness in rural Haiti.^3^

## METHODS

### Study setting.

Partners In Health, Haiti, a nonprofit organization providing health care to the poor alongside the Haitian Ministry of Health, implemented a comprehensive oral cholera vaccination campaign in the Artibonite region of Haiti between April and June 2012 in response to the ongoing cholera epidemic.^18^ A bivalent whole-cell killed OCV, Shanchol^®^, was administered to 45,417 individuals in conjunction with public health messaging about cholera vaccination and cholera prevention generated from community focus groups.^15^ Community coverage was between 76.7% and 79.2%, and 90.8% of those who received the first dose completed the full two-dose vaccination schedule.^18^ Additionally, throughout this period, multiple WASH interventions were conducted in the region by several actors as part of the national response to cholera. These interventions included distribution of water disinfection tablets, high-test hypochlorite (HTH), soap, and other supplies necessary for WASH activities, media messages to disseminate health information about cholera, promote water treatment and hygienic practices, and training of community health workers on WASH principles.^19^ Individual nongovernmental organizations constructed wells, established water purification stations, distributed water disinfection tablets, and built latrines.^20^

### Study design.

After the OCV campaign, we undertook a case-control study to evaluate the field effectiveness of OCV.^3^ To identify risk factors for cholera among cholera vaccine recipients, we conducted a case-control analysis on the subgroup of cholera cases that had been vaccinated and controls without diarrhea that had also been vaccinated. This study was undertaken in Bocozel and Grande Saline, two rural regions of Haiti. Combined, these regions have an approximate population of 55,000 people. Study participants were recruited from three health centers in the region and from the surrounding community from October 2012, 4 months after the completion of the vaccination campaign, to March 9, 2014.

### Definitions of cases and controls.

To be considered eligible for enrollment into the OCV effectiveness case-control study, cases and controls must have been residents of Bocozel or Grande Saline at the start of the study in October 2012, and eligible for vaccination at the time the campaign was conducted between April and June 2012 (age ≥ 12 months, not pregnant, and living in the region at the time).^21^ We conducted secondary analyses of data from this OCV effectiveness case-control study, restricting our analysis to cases who self-reported receiving OCV and those of their controls that also self-reported vaccination. Thus, in addition to meeting these eligibility criteria, all cases and controls included in the subgroup analysis had self-reported receiving OCV.

Cases of cholera were defined as individuals presenting with acute, watery diarrhea (three or more loose, nonbloody, liquid stools in a 24-hour period with an onset of 3 days or fewer before presentation) who had a stool sample positive for *Vibrio cholerae* by culture. Only one case per household was enrolled in the study. Controls were defined as individuals living in the community from where the cases originated, who did not present for treatment of diarrhea between the beginning of study enrollment and the date of onset of symptoms of the corresponding case. Four controls were matched to each case by location of residence, enrollment time (within 2 weeks of the case’s presentation date), and age (1–4 years, 5–15 years, and > 15 years). Controls were not matched by sex; however, an individual of the same sex was preferentially selected as a control when possible. To identify controls, study workers identified the home nearest to the case’s home, avoiding homes within the same “lakou,” a grouping of households of multigenerational families typically found in rural Haiti.^22^ Homes in the same lakou were excluded because we anticipated that exposure to cholera risk factors was likely to be highly correlated within the lakou.

### Procedures.

Stool specimens were collected in sterile containers from enrolled subjects to determine eligibility as cases. Specimens were transported in Cary–Blair media to the Haitian National Public Health Laboratory in Port-au-Prince for culture confirmation. Specimens were plated on selective thiosulfate–citrate–bile salts–sucrose agar for the isolation of *V. cholerae*. Serological confirmation was then performed using a standard slide agglutination procedure with polyvalent antisera to *V. cholerae* O1, followed by monovalent antisera to differentiate between Ogawa and Inaba serotypes.^23^

Native Haitian Creole-speaking study workers used questionnaire forms to evaluate self-reported sociodemographic characteristics, risk factors for cholera, and cholera vaccination. A description of variables included in this study is summarized in Table 1. For water treatment practices, individuals were prompted with possible options that best identified the frequency with which they treated their water; options included “always,” “almost always,” “often,” “sometimes,” and “almost never.” Enumerators also asked what methods they used to treat their water, and categorized the participants’ unprompted responses. When responses were unclear, enumerators probed for clarity. Based on prior studies and our preliminary work,^24,25^ responses were categorized a priori as follows: 1) boiling; 2) addition of household bleach such as Clorox,^®^ Jif, or granular HTH; 3) adding sodium dichloroisocyanurate tablets, Aquatabs^®^; 4) adding branded water disinfection products specifically designed for household water treatment (henceforth referred to as “branded household water disinfection products”) which included the locally marketed products Dlo Lavi^®^ (Population Services International, Pétion-Ville, Haïti), Gadyen Dlo^®^ (Deep Springs International, Léogâne, Haïti), and Klorfasil^®^ (Klorfasil, Alpharetta, GA); 5) using another water treatment method. Individuals were also asked where they primarily obtained drinking water, and these were classified into improved and unimproved sources based on criteria from the Joint Monitoring Program for Water Supply and Sanitation of the WHO.^26^ For hygiene practices, respondents were asked what type of toilet served as the main household toilet. A priori possible responses included a toilet that flushes, a toilet that does not flush, a latrine, open air defecation, or other. For cases, interviews were conducted upon enrollment in the cholera treatment unit. For controls, interviews were conducted during home visits within 14 days of enrollment of the corresponding case. Guardians or a family member proxy responded on behalf of participants younger than 18 years or unable to interview.

**Table 1:** Risk factors for cholera among individuals who received cholera vaccine in Haiti (*N* = 150)*

Variable	Cholera cases (*N* = 33)	Controls (*N* = 117)	Unadjusted relative risk	*P* value	Adjusted relative risk†	*P* value
Sociodemographic						
Female sex	10 (30)	61 (52)	0.31 (0.12, 0.84)	0.02	0.19 (0.05, 0.71)	0.01
House has earthen floor	26 (79)	85 (73)	1.82 (0.51, 6.52)	0.36		
House has electricity	4 (12)	10 (9)	3.13 (0.44, 22.40)	0.26		
Household size	5 (3, 6)	5 (4, 6)	0.96 (0.81, 1.15)	0.65		
Ever attended school	15 (45)	67 (57)	0.57 (0.24, 1.33)	0.19		
Agriculture is main income-generating activity	20 (61)	69 (59)	1.10 (0.43, 2.80)	0.85		
Household exposure and other risk factors						
Household member had diarrhea in previous week	5 (16)	15 (13)	1.31 (0.42, 4.10)	0.64		
Household member ever spent a night in cholera treatment center	10 (30)	29 (25)	1.28 (0.55, 2.97)	0.57		
Consumed food or beverage outside of household in the last week (*N* = 149)	5 (20)	23 (26)	1.21 (0.40, 3.65)	0.74		
Takes antacids (*N* = 112)	26 (81)	92 (79)	0.65 (0.21, 2.04)	0.46		
Water-related factors						
Always treats water (self-report)	11 (33)	59 (50)	0.29 (0.09, 0.96)	0.04	0.09 (0.01, 0.57)	0.01
Treats water by‡						
Boiling it	8 (24)	37 (32)	0.16 (0.013, 1.86)	0.14		
Adding household bleach (granular or liquid)	22 (67)	90 (78)	1.19 (0.31, 4.62)	0.80		
Adding Aquatabs	26 (79)	104 (90)	0.34 (0.088, 1.34)	0.83		
Adding branded household water disinfection products§	5 (15)	2 (2)	9.63 (1.11, 83.25)	0.04	44.3 (4.19, 468.05)	0.002
Using another water treatment method	9 (27)	19 (16)	0.82 (0.15, 4.64)	0.13		
Reports at least one unimproved water source as the source of household drinking water	26 (79)	93 (79)	0.81 (0.26, 2.48)	0.71		
Cholera-related knowledge						
Listed ≥ 3 ways to avoid cholera (*N* = 149)	15 (45)	52 (45)	1.00 (0.33, 3.05)	1.00		
Listed ≥ 3 ways one can get cholera (*N* = 149)	12 (36)	53 (46)	0.33 ( 0.09, 1.19)	0.09		
Listed ≥ 3 instances when one should wash hands (*N* = 149)	17 (52)	67 (58)	0.58 (0.20, 1.69)	0.32		
Sanitation and hygiene						
Has a latrine as the main household toilet	22 (67)	61 (52)	2.68 (0.93, 7.73)	0.07	4.22 (1.23, 14.43)	0.02
Washes hands ≥ 4 times per day	5 (15)	31 (26)	0.45 (0.15, 1.39)	0.16		
Vaccine-related						
Vaccine lot						
A	17 (52)	66 (56)	Reference			
B	8 (24)	35 (30)	0.86 (0.25, 2.91)	0.80		
Could not be determined	8 (24)	16 (14)	2.05 (0.70, 5.99)	0.19		

* Unless otherwise noted.

† The multivariable model included the following variables: female sex, always treats water, treats water by adding a dilute sodium hypochlorite purifier, and main toilet is a latrine.

‡ Relative to not using the referenced water treatment method.

§ Water disinfection products specifically designed for household water treatment.

### Statistical methods.

Data were entered on mobile tablets, uploaded to an online encrypted database, and analyzed using SAS version 9.3 (SAS Institute, Cary, NC). Incident cholera, indicated by case or control status, was the outcome of interest. Exposures commonly recognized as risk factors for cholera were defined as explanatory variables. We calculated matched odds ratios, 95% confidence intervals (CIs), and *P* values using univariable conditional logistic regression, adjusting for matching factors. Because we matched on broad age categories, we included age as a continuous variable in all models to account for any residual confounding by age. Risk factors for cholera at a significance level of *P* < 0.20 in the univariable analysis were included in a multivariable model and retained if the *P* value was < 0.05, the threshold for statistical significance. This conservative threshold was used to obtain a parsimonious final model, which was necessary due to the small sample size. We conducted a sensitivity analysis to examine whether the inclusion of each covariate associated with cholera with a *P* value < 0.20 but > 0.05 in multivariable analysis (i.e., other potential confounders) changed the interpretations of the risk factors reported in the final multivariable model.

### Ethics statement.

Written informed consent was obtained from all participants in this study. For those unable to consent, consent was obtained from a health-care proxy. Consent from a parent or guardian was obtained for children under 18 years of age, and assent was sought from children 7–17 years of age. All study protocols were reviewed and received ethical approval from the Partners HealthCare Institutional Review Board (Boston, MA) and the Haitian National Bioethics Committee (Port-au-Prince, Haiti).

## RESULTS

### Case-control and study population.

Of the 47 individuals with culture-confirmed cholera in the primary case control study, 33 (70%) received OCV by self-report and were included as cases in the present analysis. Of the 132 matched controls for these 33 cases, 117 (89%) reported receiving OCV and were included as controls in this analysis. Most cases and controls had received both doses of the vaccine with only three cases (9%) and 17 controls (15%) reporting receipt of one dose. Median age for cases and controls was 32 and 31 years, respectively. Additional characteristics of cases and control are reported in Table 1.

### Univariable analysis.

Univariable analysis results are summarized in Table 1. Relative to controls, cases were less likely to be female (relative risk [RR] = 0.31, 95% CI = 0.12–0.84, *P* = 0.02). Overall, the frequency of self-reporting always treating water was low among both cases (33%) and controls (50%), and cases were less likely to report always treating their water (RR = 0.29; 95% CI = 0.09–0.96, *P* = 0.04) compared with controls. Among the different methods of treating water, self-reporting use of branded household water disinfection products as a treatment method was significantly higher among cases (15%) than controls (2%) (RR = 9.63, 95% CI = 1.11–83.25, *P* = 0.04). Self-reporting having a latrine as a main toilet as compared with practicing open-air defecation was common among both cases (67%) and controls (52%), with cases tending to be more likely to report having a latrine as the main household toilet (RR = 2.68, 95% CI = 0.93–7.73, *P* = 0.07). No respondents reported having a flush toilet. Among those who had a latrine as the main household toilet, the median number of people with whom they shared it was 15 (interquartile range [IQR] = 8–26).

### Multivariable analysis.

The final multivariable model included the following variables: age, female sex, household self-reporting always treating their water, treating water by adding a branded household water disinfection product, and having a latrine as the main household toilet. Reporting the use of branded household water disinfection products as a means of treating water, relative to no use (adjusted relative risk [aRR] = 44.3, 95% CI = 4.19–468.05, *P* = 0.002), and having a latrine as the main household toilet (aRR = 4.22, 95% CI = 1.23–14.43, *P* = 0.02) versus open-air defection were independent risk factors for cholera. Female gender (aRR = 0.19, 95% CI = 0.05–0.71, *P* = 0.01) and self-reporting always treating water (aRR = 0.09, 95% CI = 0.01–0.57, *P* = 0.01) were associated with protection against cholera. Individuals that reported using a branded household water disinfection product reported always treating their water with a frequency of 57.1%, compared with a frequency of 46.5% among those that did not report using this method to treat their water (Fisher’s exact *P* value = 0.71).

Other risk factors that met criteria for inclusion in the multivariable model (*P* < 0.20) such as having ever attended school, treating water by boiling it, using another water treatment method (alternative methods of treating water such as filtration, addition of lemon or citrus, flocculation with the cactus *Opuntia tuna*, PuR^®^ sachets, or adding salt and solar disinfection), correctly listing more than three ways one can get cholera, and handwashing more than four times per day were not statistically significant in multivariable analyses and were therefore excluded from the final multivariable model. In the sensitivity analysis in which we singularly adjusted for each of these variables, we found no change in directionality or statistical significance to any of the variables included in the final model.

## DISCUSSION

Our study highlights risk factors for cholera in a high-risk rural population that has received OCV, as well as some, but neither complete nor systematic water and sanitation interventions.^19,20^ We found that among individuals vaccinated against cholera, consistent water treatment was a key factor in reducing the risk of cholera. Having a latrine as the main household toilet, as opposed to practicing open-air defecation, was a significant risk factor for cholera among this group. Our results also show that those who reported treating water with branded household water disinfection products had an increased risk of cholera. These findings have important implications for policy-makers in Haiti, and other regions where cholera epidemics occur.

Open-air defecation is associated with an increased risk of surface water contamination with diarrheal pathogens such as *V. cholerae*, and the reduction of open-air defecation is an important public health priority for communities.^27,28^ As such, improving access to latrines is a critical part of improving sanitation and reducing the burden of diarrheal disease.^29^ However, we found that having a latrine as the main household toilet, as opposed to practicing open-air defecation, was associated with an increased risk of cholera for individual participants. This finding has been previously reported in Haiti^30^ and elsewhere.^31,32^ Similarly, a study conducted in Bangladesh demonstrated an increased risk of pediatric shigellosis associated with the presence of a family latrine, and interestingly, removal of unsanitary latrines decreased the risk of pediatric shigellosis.^33^ We did not directly inspect latrines, so it is not possible to know from our study if this elevated risk is a result of poorly maintained latrines, or whether the increased risk reflects poor hygiene practices, or another unmeasured variable. The increased risk may be partly explained by latrine sharing. In our study, among households that had access to latrines, the median number of people with whom the respondent shared the latrine was 15 (IQR = 8–26). Concerns surrounding the cleanliness, accessibility, and potential for negative health outcomes of public or shared latrines has led the Joint Monitoring Program for Water Supply and Sanitation of the WHO to classify otherwise improved sanitation facilities that are shared by multiple households as unimproved facilities.^28^ Recent studies comparing the risk of diarrhea associated with individual household latrines compared with communal latrines or latrines shared by multiple households have also demonstrated an increased odds of diarrhea associated with latrine sharing.^34,35^ A recent analysis from the Global Enteric Multicenter Study demonstrated similar findings, and showed that exposure to fecal contamination in shared latrines was also associated with an increased risk of diarrhea among children at certain sites.^36^ It is also possible that the increased risk of cholera among individuals self-reporting having a latrine as the main household toilet was a result of fecal contamination of nearby water sources from inappropriate construction and maintenance of latrines, and that this contamination disproportionately affected latrine owners. This has previously been documented, although we did not evaluate the distance between latrines and water sources as part of this study.^37^ Universal access to safe water and sanitation is a human right, this is not in doubt. However, further investigation is needed to understand what sanitation measures constitute key priorities for policy-makers desiring to control cholera transmission. Our findings also caution against counting of “latrines built” or “open defecation free communities” as a measure of progress towards elimination of transmission of cholera, but rather would support the need for effectiveness measures for these interventions.

The protective effect of consistently treating drinking water in our study was not surprising. However, our finding that individuals who reported treating water with a branded household water disinfection product had a significantly elevated risk of contracting cholera was unexpected. The small number of observations in this category suggests that these data should be interpreted with caution as this may be a chance finding. These products included water disinfection products locally marketed and/or distributed throughout Haiti for household water treatment, branded as Dlo Lavi^®^, Gadyen Dlo^®^, and Klorfasil^®^. The risk associated with reporting the use of these water disinfection products in our study was unrelated to frequency of use – people who used these products self-reported always treating water with a frequency of 57%, as compared with 46% among those who did not use these products. Regardless, we adjusted for frequency of treating water in our multivariable analyses, ensuring that the increased risk of cholera associated with reporting use of these products was independent of the frequency of treating water (i.e., the lack of effectiveness of the category was not that people reported using the products less frequently than other categories).

It is possible that the increased risk seen with using branded household water disinfection products in our study represents improper use. Previous studies in Haiti highlighted a low rate of detectable chlorine levels in stored drinking water despite the availability of chlorine products, suggesting improper or insufficient use of these products, or suboptimal product quality^38^ and highlighting the importance of appropriate instruction, and a consistent, affordable supply chain.^24,30^ Other studies in this region showed that although most people reported treating their water in the last 3 months, only a small percentage actually had a water treatment product present at the time of a home visit,^24^ and that the most common reason for not always treating water was lack of access to products.^15^ Cost and limited access to water treatment products also remain significant barriers to appropriate household water treatment in this region.^15,24^ The turbidity of source water, known to be high in the region where the study took place, may have also contributed to diminished efficacy of these products, since turbid water should usually be filtered before standard doses of chlorine can be applied with appropriate effect.^39^ Another possibility is that the emergence of increasing bacterial resistance to chlorine may also contribute to the reduced efficacy of branded household water disinfection products, relative to other water treatment methods. Because we did not sample household water supplies to test for free residual chlorine or conduct microbiologic testing of water samples as part of our study nor view chlorine products in the household, we cannot determine whether one of these possibilities, or chance, contributed to this finding. Our study does not call the efficacy of these products into question. However, the fact that these specific branded household water disinfection products increased the risk of cholera in this study raises concerns about their field use and suggests potential failures in implementation. Notably, other household water treatment programs in Haiti with sufficient training, follow-up, and supply chain have shown better results, and should be explored as models of field effectiveness.^40–42^ Concerns about implementation highlight the fact that efficacy does not always translate to impact,^43^ and our findings also support recent systematic reviews that suggest an urgent need for further studies to evaluate the field effectiveness of household water treatment options, and their impact on cholera control.^44,45^

We also found that female sex was a significant protective factor for cholera among our cohort (i.e., men were over-represented among cases). This finding has never been documented to our knowledge, nor have previous studies demonstrated sex-specific differences in immune responses after cholera vaccination. Approximately 50% of controls in the study were men, representing the sex distribution in rural Haiti, so this finding is unlikely to be an artifact of control selection. There are at least two possibilities that would explain why men are overrepresented among cases: 1) there may be one or more unmeasured risk factors for cholera that are more common in men in this setting; 2) men may be more likely than women to seek care for their diarrhea, and therefore more likely to be identified as a cholera case in this study.

There are some limitations associated with this study. It is possible that some of our controls had asymptomatic cholera. This would attenuate the effect estimates for risk factors common to symptomatic and asymptomatic cholera. Although this study only evaluates risk factors among symptomatic cases of cholera, asymptomatic cholera is a significant public health risk as these individuals are still infectious.^46^ It is unclear to what extent vaccination against cholera prevents asymptomatic disease and transmission. We cannot rule out differential recall of exposures among cases and controls. However, the data suggest that recall bias is an unlikely explanation of our findings. For example, having a latrine as the main household toilet was not associated with noncholera diarrhea.^3^ This provides strong evidence against recall bias because participants did not know whether their illness was due to cholera, versus some other etiology, at the time of interview. If present, we would expect to see recall bias among both cholera and noncholera diarrhea cases. We did not compare risk factors for cholera among the vaccinated to risk factors for cholera in the unvaccinated because the number of subjects was too small to have sufficient power. However, given that millions of doses of OCV have been deployed, and scale up is ongoing globally, we believe that there is sufficient importance in understanding the risks in vaccinated populations to merit the study as described. Of the controls included in the analysis only 15% received one dose of OCV and thus may not be protected against cholera to the same degree as individuals receiving both doses. We believe their inclusion is justified because the primary case-control analysis found similar vaccine effectiveness among individuals receiving one dose and those receiving two doses.^3^ Additionally, large-scale risk factors associated with cholera outbreaks such as changing weather patterns, human migration patterns, and variations in plankton ecosystems, are beyond the scope of this study. Finally, lack of specific detail on how regional WASH programs were implemented at the individual and household level means that we cannot attribute our findings to any particular aspect of the implementation. However, our findings suggest that a more detailed evaluation of household WASH interventions’ impact on cholera control in the region is justified.

This study represents the first evaluation of risk factors for cholera among OCV recipients. WASH remains a key factor for cholera control in the current protracted epidemic setting of Haiti, even among vaccinated individuals. Our findings reinforce the critical importance of pairing cholera vaccination campaigns with efforts that deliver effective safe water solutions. They also highlight the importance of measuring the field effectiveness and impact of WASH interventions on cholera control, rather than assuming that the quantity of latrines constructed and quantity of household water treatment products delivered will result in disease control.
